# A Review of the Diagnosis and Treatment of Ochratoxin A Inhalational Exposure Associated with Human Illness and Kidney Disease including Focal Segmental Glomerulosclerosis

**DOI:** 10.1155/2012/835059

**Published:** 2011-12-29

**Authors:** Janette H. Hope, Bradley E. Hope

**Affiliations:** ^1^Environmental Medicine, 304 West Los Olivos Street, Santa Barbara, CA 93105, USA; ^2^American Indian Health and Services, 4141 State Street B-4, Santa Barbara, CA 93110, USA

## Abstract

Ochratoxin A (OTA) exposure via ingestion and inhalation has been described in the literature to cause kidney disease in both animals and humans. This paper reviews Ochratoxin A and its relationship to human health and kidney disease with a focus on a possible association with focal segmental glomerulosclerosis (FSGS) in humans. Prevention and treatment strategies for OTA-induced illness are also discussed, including cholestyramine, a bile-acid-binding resin used as a sequestrant to reduce the enterohepatic recirculation of OTA.

## 1. Introduction

Ochratoxin A (OTA) is a known nephrotoxic, immunotoxic, and carcinogenic mycotoxin in animals [1, 2] that has long been studied for its role in animal and human disease. Molds associated with the production of OTA include *Aspergillus ochraceus, Aspergillus niger *and* Aspergillus carbonarius, Penicillium verrucosum*, and species of *Penicillium, Petromyces,* and *Neopetromyces *[3]. Concerns regarding exposure to ochratoxin have primarily centered on exposure to food contaminated with OTA such as wine, beer, coffee, dried vine fruit, grape juices, pork, poultry, dairy, spices, and chocolate [1]. Toxicity from ochratoxin is considered serious enough that it is among approximately 20 mycotoxins monitored in food.

 Options for diagnosis and treatment of persons diagnosed with chronic kidney disease and FSGS are reviewed. Treatment is then discussed in the context of two patients diagnosed with FSGS who were found to have significantly elevated levels of urine OTA. This exposure is believed to have resulted from inhalational exposure from water damaged indoor environments found to have elevated levels of mold including species of *Aspergillus *and *Penicillium.* This review is intended to highlight the importance of prevention and treatment of human kidney disease and ochratoxin exposure from indoor mold.

## 2. Mechanisms of Toxicity

Ochratoxins occur in nature as Ochratoxin A, B, and C. OTA is the most prevalent toxin, and our discussion will be limited to OTA. OTA is a proven carcinogen in animals and is classified as a class 2B, possible human carcinogen by the International Agency for Research on Cancer [4]. The National Toxicology Program (NTP) has designated OTA as “reasonably anticipated to be a human carcinogen” based on sufficient evidence of carcinogenicity in experimental animals [1].

After initial exposure from any source, the urinary and fecal excretory routes of OTA are both important with the relative contribution of each dependent upon factors such as route of administration and dose [5]. In the blood, OTA binds to albumin and the bound fraction constitutes a mobile reserve of OTA [6]. The relative contribution of each excretory route is influenced by the degree of serum macromolecular binding as well as differences in the enterohepatic recirculation of OTA [7]. Elimination of OTA in urine and feces is felt to be relatively slow and has been shown to vary by species and gender as well as specific genotype that may affect the biotransformation of OTA [8, 9].

Intestinal microflora also appear to contribute significantly to the metabolism of OTA via hydrolyzation to the less toxic ochratoxin alpha in rats [10]. Inhibition of microflora in the lower GI tract of rats by neomycin results in decreased hydrolysis of OTA to ochratoxin alpha resulting in elevated levels of OTA [11]. In addition, administration of radiolabeled OTA to rats indicated that effective metabolism of OTA was lacking in most tissues other than the intestines [12]. The importance of digestion in the detoxication of OTA is also supported by the observation that OTA does not readily accumulate in ruminants due to rapid detoxification in the extensive ruminant stomach [5, 13].

Limited information is available on the metabolic disposition of OTA in humans, although it has been suggested that it has a long serum half-life due to strong binding to human serum macromolecules [7, 14].

Individual genetic differences affect the biotransformation and relative toxicity of OTA, with enzymatic hydrolysis and cytochrome p450 induction felt to play a role in toxicity. Studies have indicated that the biotransformation of OTA can be effected by CYP 3A4, CYP 1A1, and CYP 2C9-1 while conflicting results have been found for CYP 1A2 [15, 16].

 DNA adducts also occur in animals exposed to OTA in all available studies [17–20]. DNA adducts consist of a chemical covalently bound to DNA. This could interfere with the DNA repair systems and cell cycle controls systems and serve as an initiating point of carcinogenesis.

Oxidative stress is another component of OTA toxicity [21]. Pretreatment of rats with retinol (vitamin A), ascorbic acid (vitamin C), or alpha tocopherol (vitamin E) before OTA administration significantly decreased the number of DNA adducts formed in the kidney by 70 percent, 90 percent, and 80 percent, respectively [22]. In addition, lipid peroxidation and enzymes involved in arachidonic acid metabolism affect the biotransformation of OTA [23].

More recently, it is been shown in rodents that mTOR/AKT pathways are significantly deregulated after exposure to OTA, possibly contributing to carcinogenicity in kidney cells [24].

## 3. Tissue Distribution

Tissue distribution after exposure of animals to OTA has consistently revealed that the greatest concentration is in the kidneys followed by either liver or muscle and then fat [25–27], but tissues found to contain OTA also include the adrenal medulla and cortex, skin, myocardium, gastric mucosa, and bone marrow [28].

In humans, OTA has been detected in blood, urine, and breast milk [29–31] as well as renal cell carcinomas, breast cancer, astrocytoma, inflamed bladder tissue and transitional cell carcinoma of the bladder, and a skin biopsy sample [32]. Recently, a case has been reported of OTA being found in the umbilical cord and placental tissue of a newborn whose mother had been exposed from a water-damaged home. In addition, the mother had OTA in her breast milk, urine, and nasal secretions [33]. Also, other family members tested positive for OTA in urine and nasal secretion samples, while the pet dog was positive for OTA in its urine and an ear mass [33].

## 4. Agents Modulating Toxicity

Various substances have been found to either increase or decrease the toxicity of OTA. In mice, pretreatment with phenobarbital decreased the toxicity of OTA with significant increases in LD50 seen [34, 35]. Administration of piperonyl butoxide was also shown to significantly decrease the LD50 of OTA [35] thus increasing toxicity.

A protective effect of melatonin and licorice plant extract was demonstrated in rats exposed to OTA for 28 days and alleviated most of the biochemical abnormalities associated with the exposure [36]. It is significant in this study that the histopathological abnormalities were seen in this relatively short 28 day exposure and showed degenerative symptoms in the proximal tubules, congestion in renal tissue, and a remarkable infiltration of inflammatory cells consistent with OTA nephropathy.

Phenylalanine prevents acute poisoning by OTA in mice [37]. Aspartame, a structural analogue of phenylalanine, is also a powerful inhibitor of OTA toxicity, at least in animals [38].

In male rats, OTA was more toxic in the presence of phenylbutazone (a nonsteroidal anti inflammatory drug/NSAID) and ethyl biscoumacetate (vitamin K antagonist/coumarin) and was less toxic when administered with sulfamethoxypridazine (a sulfonamide antibiotic) [39].

## 5. Exposure to Ochratoxin A from Food

Dietary exposure to OTA has been extensively documented in the literature and worldwide remains a significant source of OTA exposure in humans [1, 4]. Background studies on average levels of OTA in humans eating a typical diet have, however, only shown modest elevations in urinary OTA levels, well under the 2.0 ppb limit of detection level used by the commercial lab to test these patients [40].

## 6. Inhalational Exposure to Ochratoxin A

Throughout the literature, much of the study of ochratoxin exposure focuses on exposure through ingestion. However, inhalation exposure in water-damaged buildings with amplified indoor growth of ochratoxin producing species of mold remains a significant risk. OTA has been identified in studies of water-damaged buildings from air [41], dust [42], wallpaper [43], and agricultural dust and conidia [44]. Hooper et al. have reported elevated concentrations of OTA in the urine of individuals exposed to water-damaged buildings versus unexposed controls [40]. The concentrations of mycotoxins for controls not exposed to water damaged buildings were below the detection limit which is 2.0 ppb for ochratoxin [40].

In a study by Skaug et al., dust and aerosol samples were collected from three Norwegian cowsheds [44]. OTA was detected in 6 out of 14 samples with concentrations ranging from 0.2 to 70 *μ*g/kg (ppb) [44]. Collected conidia also contained OTA. The authors concluded that airborne dust and conidia can be sources of OTA and that peak exposures and absorptions from this route can be considerable, especially given the efficiency with which OTA is absorbed through the lung [44, 45]. Testing has also indicated the presence of OTA from samples of air filters, refrigerator filters, dust from air vents, and a towel and sandals from water-damaged buildings [32, 33].

In another study of dust collected from heating ducts in a household where animals were exhibiting signs of ochratoxin poisoning, it was found that all samples in one group yielded ochratoxin, with samples from one duct being over 1500 ppb and another duct showing levels of 306 ppb. In a sample group in the same study, the dust from six samples was measured in a composite showing a level of 58 ppb [42]. In a subsequent study using high-performance liquid chromatography to quantify airborne mycotoxins in a poultry house in Dalian, China, OTA along with aflatoxin and zearalenone was detected [41].

Rapid systemic appearance of OTA after inhalation exposure in rats has been documented with a 98 percent bioavailability [45]. This clearly makes inhalational exposure to OTA a very significant risk.

One of the most striking cases documented in the literature involves the onset of acute renal failure from inhaled OTA after an 8-hour exposure to a granary that had been closed for several years in which an ochratoxin-producing strain of *Aspergillus ochraceus* was isolated [46]. The couple who were exposed to this granary experienced respiratory distress, retrosternal burning, epigastric tension, and asthenia. The husband's condition improved within 24 hours; however, the women's condition worsened and she was admitted 5 days later with nonoliguric renal failure, pulmonary edema, periorbital and lower extremity edema, and proteinuria of 4.6 g/L. A biopsy showed acute tubulonecrosis with interstitial edema with localized infiltration of lymphocytes, granulocytes, and macrophages with thickening of the basement membrane. Fortunately, her kidney function returned to normal 40 days after the exposure.

## 7. Ochratoxin and Renal Disease

Ochratoxin A has been found to be nephrotoxic in all mammalian species treated [6], although differences in toxicity have been found among species and sex. OTA is excreted in both the stool and urine. Although likely a minor route of excretion, ochratoxin has also been found in human sweat [47].

OTA has been associated with human kidney disease and is the probable causative agent of Balkan Endemic Nephropathy (BEN) [48], a severe, progressive, and ultimately fatal renal disease affecting populations in the Balkan Peninsula. Studies have indicated elevated levels of OTA in persons affected by BEN compared to neighboring persons in unaffected Bulgarian villages [1]. Additionally, several studies in animals have confirmed a causal connection between OTA exposure and cancers of the urinary tract, liver, and mammary glands [49–52]. Striking similarities have been noted between OTA-induced porcine nephropathy in pigs and BEN in humans [21].

The main nephrotoxic effect is in the postproximal nephron and proximal tubule which have been reported as a self-enhancing effect [53, 54].

A recent study in Sri Lanka measuring mycotoxin levels in the urine of patients with kidney disease demonstrated the presence of ochratoxin in 93.5 percent of patients tested although ochratoxin was also found in individuals without kidney disease [55]. Clark's review of ochratoxin in the blood concluded that the highest levels were observed in sampled populations that included persons with kidney disorders with serum level as high as 35–100 ng/mL (ppb) [1]. In contrast, mean serum OTA level in Europe was found to be 0.1–2 ng/mL (ppb) [1].

A correlation of consumption of foods known to contain OTA and the incidence of testicular cancer in 20 countries has suggested the possibility of OTA being related to an increased incidence of testicular carcinoma. The authors also report that there is correlation of pork and coffee intake with testicular carcinoma. In addition, animals exposed to OTA contain OTA in the testes and OTA causes adducts in testicular DNA [56].

Several studies on Tunisians with and without renal disease have shown elevations in serum OTA levels in both populations, with higher levels being found in those with renal disease. In one report, the mean value of OTA for the healthy control population was 3.3 ± 1.5 ng/mL (ppb) compared to a mean value of 18 ± 7 ng/mL (ppb) in those with chronic interstitial nephropathy of unknown origin [57]. Another study of OTA in human blood samples comparing persons with various types of chronic kidney disease to controls showed elevations in serum ochratoxin which were greatest in those diagnosed with chronic interstitial nephropathy at mean values of 25–59 ng/mL (ppb) compared to 0.7–7.8 ng/mL (0.7–7.8 ppb) in the general population and 6–18 ng/mL (ppb) in those with other types of kidney disease [58].

## 8. Ochratoxin and Reproduction

Ochratoxin can cross the placenta and has been found to be embryotoxic in rats and mice [6]. Studies with radiolabeled OTA in mice showed OTA to cross the placenta [59], preferentially at specific times during gestation. Additionally OTA has been found in breast milk which could represent a significant source of exposure for infants [33, 60].

A study of rats exposed to OTA preconception, during gestation and during lactation showed that the exposed offspring had three to four times higher levels of OTA than the controls. The rat offspring exposed to OTA in both in utero and through breast milk were found to have the highest blood and kidney concentrations of OTA, with the most significant exposure attributed to lactation [60]. In this study, the transfer of OTA to breast milk was found to be very efficient with levels at 60 percent of blood concentrations. Moreover, a study in rabbits showed an effective transport of OTA from blood to milk and subsequently to the offspring with plasma and kidney concentrations much higher in offspring than adults, possibly due to slower detoxification in the offspring [61]. A study involving 80 Norwegian women found that 21 percent of the breast milk samples showed elevations of OTA ranging from 10 to 182 ng/L (ppt) [62]. The authors believed that these observations were very significant since studies in neonatal rats have shown that neonates are much more susceptible than adult rats with LD50 values for OTA from the oral route being only 3.9 mg/kg in neonates compared with adult LD 50 values of 20–330.3 mg/kg in adults [5]. In addition, testing breast milk samples from 75 women in Ankara, Turkey whose children were patients in the Neonatology Department, showed OTA in all samples tested in the range of 620.87–11311.30 ng/L (ppt) [63]. Additionally, OTA concentration in fetal serum was reported to be twice that of the mother indicating an active transfer of OTA across the placenta [64].

OTA was also shown to decrease testosterone secretion in testicular interstitial cells of gerbils [65].

## 9. Ochratoxin and the Brain

*In vitro *and *in vivo *research has demonstrated cerebellar [66, 67], hippocampal [68], and other adverse neurological effects due to OTA [66–69]. A single dose of OTA to Swiss mice was associated with significant oxidative damage in six brain regions—the cerebellum, hippocampus, caudate putamen, pons medulla, substantia nigra, and cerebral cortex. Peak effects were observed in the midbrain, caudate/putamen, and hippocampus [69]. In addition, striatal dopamine was decreased after a single exposure to OTA [69]. *In vitro *experiments have shown decreased proliferation of neural progenitor stem cells in the hippocampal region of mice after exposure to OTA leading the authors to speculate that problems impairing hippocampal neurogenesis *in vivo* could contribute to the memory problems and depression commonly seen in humans exposed to mycotoxins [68]. In another study evaluating the neurotoxicity of ochratoxin A, primary neurons and neuronal cells were incubated with increasing concentrations of OTA [66]. A dose-dependent increase in cytotoxicity was found in both cell types resulting from apoptosis and accompanied by a loss of mitochondrial membrane potential [66]. Based on these data, the authors speculated that OTA may contribute to the development of neurodegenerative diseases such as Alzheimer's and Parkinson's in which apoptotic processes are centrally involved [66].

## 10. Ochratoxin and Immunity

OTA is known to be immunotoxic in animal studies [1, 2, 70]. The immunosuppressant activity of OTA in animals has been characterized by size reduction of vital immune organs like the thymus, spleen and lymph nodes, depression of antibody responses, alterations in the number and functions of immune cells, and modulation of cytokine production [70].

There are also complex relationships between *Aspergillus*, T regulatory lymphocytes and candidiasis [71], which can be clinically relevant in humans.

## 11. Focal Segmental Glomerulosclerosis (FSGS)

FSGS is a potentially devastating kidney disease that occurs most frequently in children and young adults but can occur at any age. In early stages of the disease, the kidneys are typically normal or enlarged, while in the late stage of the illness, kidneys are typically shrunken [72].

 FSGS accounts for approximately one sixth of the cases of nephrotic syndrome in children and is a common cause of kidney failure in adults. The annual incidence of end-stage renal disease from FSGS has increased 11-fold from 0.2 percent to 2.3 percent between 1980 and 2000, and FSGS is now the most common cause of end-stage renal disease resulting from primary glomerular disease [72].

There has been increasing recognition of causes in primary FSGS including genetic, viral, drug toxicity, and others. Cases are considered primary with no cause identified or secondary due to infections (HIV, Hep B, Parvovirus), toxins (heroin, pamidronate, analgesics), familial, or nephron loss from chronic pyelonephritis, obesity, diabetes, sickle cell disease, or anatomic abnormalities and malignancies [71]. It is not uncommon for the onset of illness to occur after an upper respiratory infection. Ethnic differences are seen in the prevalence of the disease with blacks affected seven times more often as whites with a worse prognosis once the disease is acquired [72].

Treatments for FSGS include salt and protein restriction, diuretics for edema, ACE inhibitors, aldosterone antagonists, steroids, cytotoxics agents (e.g., cyclophosphamide), immunosuppressants (e.g., cyclosporin and tacrolimus), plasmapharesis, and treatment for hyperlipidemia which commonly occurs with the illness [72]. Rarely intravenous albumin or mannitol has been used for intractable edema. Often no treatment is successful and the patient requires dialysis and eventual transplantation. In patients who do not respond to treatment, the average time from onset of the disease to end-stage renal disease is from 6 to 8 years [72].

Recurrence of the disease posttransplantation has long been recognized as a significant risk. In one study of 77, mostly pediatric patients with idiopathic nephrotic syndrome and FSGS who underwent transplantation, 42 had nephrotic range proteinuria posttransplant, and 20 eventually developed pathology consistent with FSGS in the transplanted kidney [73]. Interestingly, the majority of the recurrence occurs in the first 6 months, with the recurrence of illness rare after two years posttransplantation.

## 12. Human FSGS and Kidney Disease Associated with Ochratoxin A: Patient Histories

Two patients histories of FSGS associated with OTA exposure encountered by the authors are presented.

The first patient was a 48-year-old woman diagnosed with primary idiopathic FSGS who presented with end-stage renal disease 10 days before a scheduled kidney transplantation. She had been referred by her daughter's pediatrician after she expressed concern about her daughter's serious respiratory symptoms and asthma exacerbations and indicated that her home had recently been found to have significant mold contamination including elevations of *Aspergillus/Penicillium *and *Stachybotrys *on nonviable spore sampling. The most significant elevations were found in the room she had used as a home office. The patient worked as a marriage and family therapist from her home office until she was no longer able to work due to her illness. She moved into the home 12 years prior to presentation and believes the water damage to have been long standing, predating her move. Shortly after moving into the home she developed symptoms of chronic fatigue syndrome which have persisted. Three and a half years before her presentation to the office, she was seen in the emergency room for severe edema and diagnosed with nephrotic syndrome. The diagnosis of FSGS followed several kidney biopsies after it was initially undetected. Attempts to control the disease with long-term high-dose steroids were unsuccessful, and the disease progressed to the point where she was placed on peritoneal dialysis and the search for a donor kidney commenced. At the time of presentation to this office she was taking vitamin D, folic acid, dialyvite, calcitriol, sevelamer carbonate (a phosphate binder), and rosuvastatin for hyperlipidemia. Hyperlipidemia is a common complication of her type of renal disease or nephrotic syndrome. Physical exam was remarkable for a tired, uncomfortable appearing woman who felt nauseated on several occasions during the exam. She displayed 1+ pitting pretibial edema, mucosal nasal swelling with white patches noted, a thick white coating on her tongue, a I/VI systolic murmur which did not radiate, and moderate dysmetria on finger to nose testing. Urine mycotoxin testing was performed, and the patient was found to have significant elevations of ochratoxin A at 11.9 ppb (limit of detection 2.0 ppb). Aflatoxin and trichothecene mycotoxins were tested and were not detected. The testing for ochratoxin A was performed by a CLIA certified lab using immunoaffinity columns and fluorometry [40]. The patient reported a history of recurrent yeast infections necessitating the use of oral fluconazole and was found to be anergic to candida on intradermal skin testing, with a normal response to tetanus noted. A nasal fungal culture showed the presence of *Cladosporium *species, while fungal blood cultures remained negative. Testing for antibodies to *Aspergillus* species of mold was recommended but not performed. Prior to the dietary changes required by the onset of renal failure, the patient's diet was typical of the general population and did not include excessive consumption of foods known to contain OTA.

The patient was encouraged to commence treatment with oral cholestyramine as soon as possible, but met with resistance from her transplant team and ultimately did not initiate therapy. She did, however, follow the recommendation to move from her home to which she has not yet returned and has avoided further exposure to items exposed to the mold contaminated home. Her posttransplantation course was remarkable for the recurrence of proteinuria within days of the transplantation. Her function in the transplanted kidney has continued to deteriorate, and she was diagnosed with FSGS in the previously healthy donor kidney within months of the transplantation. Additionally, she has experienced rejection of the transplanted kidney requiring a course of solumedrol and long-term use of immunosuppressant agents. To date, the patient has not undergone any treatment to reduce the body burden of ochratoxin indicated by her initial elevations in urinary ochratoxin other than avoidance of her water-damaged home.

The second patient was a 5-year-old girl who presented to the office with a history of receiving a diagnosis of FSGS at the age of 3.5 after proteinuria was identified when she presented to her pediatrician experiencing new onset enuresis. The family sought care from the authors due to chronic symptoms and illnesses in all family members and history of exposure to water-damaged environments in past and current homes. Due to the father's work, the family has moved frequently. They recall evidence of water damage and mold in several of their previous homes and confirmed the presence of elevated levels of indoor mold in their current home. The patient's diet was typical for her age and did not include excessive consumption of foods known to contain elevated levels of ochratoxin.

Testing indicated the patient did not have a genetic cause for FSGS. Upon diagnosis she was placed on a 6-week course of high-dose prednisone and has since used tacrolimus and enalapril as well as galactose. Her protein excretion in urine is followed regularly, and her mother reports an increased level of proteinuria upon moving to their most recent home.

She was born by NSVD at 39 weeks gestation. APGAR scores are not available. Her mother reports she required some initial resuscitation, but improved quickly and did not require transfer to neonatal intensive care unit. At 5 days of age, she was found to have severe hyperbilirubinemia, with reported levels of 26 mg/dL. She was breastfed for 14 months.

Her physical exam was remarkable for nasal mucosal swelling with clear discharge and erythematous right tympanic membrane consistent otitis media, slightly enlarged bilateral submandibular lymph nodes, and moderate sway when balancing on toes with eyes open and an inability to balance on toes with eyes closed.

 Urine mycotoxin testing was performed, and she was found to have significantly elevated level of ochratoxin A at 9.1 ppb (limit of detection 2.0 ppb). Aflatoxin and trichothecene mycotoxins were not detected in the sample. Of note, elevations in urinary levels of ochratoxin and trichothecene mycotoxins were found in other family members as would be expected with their shared inhalational exposure; however, the patient's level of OTA was significantly higher than that of the other family members.

At the time of presentation to this office, the patient was also reporting symptoms of night sweats, heat intolerance, frequent episodes of otitis media and conjunctivitis, dizziness, hair loss, fungal skin rashes, episodes of excessive thirst, and the recent onset of reversing letters when she writes. Additionally, she has a history of significant dental disease including requiring a root canal at the age of 4.

The patient was started on therapy including avoidance of exposure to water-damaged/moldy environments and property exposed to these environments and the use of nutritional support, liposomal glutathione, and sequestering agents including cholestyramine and charcoal. Within months of starting treatment, the mother reported a significant decrease in the patients urinary protein excretion with the lowest levels found to date on recent testing.

## 13. Treatment

Given the well-known nephrotoxicity of OTA, as well as reports of human kidney disease associated with elevated levels of OTA, including BEN and other kidney disease, and this report of elevated OTA in two patients diagnosed with FSGS, we believe it is appropriate to obtain a detailed clinical history documenting potential exposures to water-damaged buildings with elevated levels of mold as well as a dietary history of persons diagnosed with FSGS and significant renal disease for which an alternative explanation is not readily available. Since testing for mycotoxins in urine and other tissues is now readily available [40], it is appropriate to obtain this testing on urine and renal biopsy tissue to help elucidate whether this is a potential contributor to illness. In cases where elevated levels of OTA and/or evidence of significant indoor water damage is found, urging avoidance of further exposure would be strongly recommended. In addition, in cases where the disease has progressed to the state where kidney transplantation is indicated, knowing if the existing kidneys have a significant concentration of OTA might affect the decision about whether to leave the diseased kidneys in place at the time of transplantation.

Various other means of lowering the renal burden of OTA have been studied with varying degrees of success. Most promising is the use of cholestyramine as a bile acid resin binding agent to reduce enterohepatic recirculation of OTA, thereby reducing levels that are filtered through the kidneys and shifting excretion to the stool where it is presumably bound to cholestyramine resin. Cholestyramine is not absorbed systemically allowing it to be safe even for those with advanced kidney disease. Studies in rats showed that OTA exposed rats that were fed a diet enriched with cholestyramine experienced decreased OTA concentration in plasma as well as decreased excretion of OTA and its metabolites (ochratoxin alpha and hydroxylated ochratoxin A) in bile and urine [74]. This was associated with an increased excretion of OTA in feces which was felt to reduce the potential nephrotoxicity of OTA.

Studies of sweat have shown the presence of OTA in sweat on at least one occasion [47] supporting treatments such as sauna to increase excretion of OTA.

Several studies have indicated that phenylalanine decreases the absorption and consequent toxicity of OTA, and this is also true of aspartame [34, 37, 38].

## 14. Discussion

We have reviewed two cases of FSGS associated with significant elevations in urinary OTA excretion following inhalational exposure in water-damaged buildings. The nephrotoxicity of OTA is undisputed in the literature in both extensive animal studies and more limited human studies. The precise role, if any, ochratoxin plays in the onset and progression of FSGS in select individuals has yet to be elucidated.

The etiology of FSGS is generally unknown, while treatments are most often directed towards slowing progression. At least in the case of drug toxicity including heroin and other drugs, toxins have been acknowledged to play an important role in some cases of FSGS. Undoubtedly, like most human illnesses, it will involve a complex interaction of environmental exposures with underlying genetics. Further study is clearly indicated; however, in the interim, it is wise to test for and initiate appropriate, safe measures to lower the body burden of OTA. As has been discussed, a toxin as potent as OTA that is known to be found in the highest concentrations in kidney tissue could certainly play a role in the onset and progression of the disease. A kidney already damaged from any cause would be all the more negatively affected by the strain of excreting a potent nephrotoxin.

It is the recommendation of the authors to obtain a detailed environmental history including exposure to water-damaged indoor environments and a dietary history in the case of primary idiopathic FSGS as well as other kidney disease of unclear etiology. If the history is remarkable for any potential exposures to OTA and perhaps even if it is not, we would urge evaluation for the presence of OTA in urine. If biopsy samples are available, it would also be useful to test these tissues since elevated levels in the kidney, might support the removal of diseased kidneys at the time of transplantation as opposed to the common practice of leaving them in place. In light of the detection of OTA in individuals with chronic fungal sinusitis [33], testing of nasal secretions for OTA and other mycotoxins should also be considered.

Additionally, consideration should be given to the testing of breast milk for OTA and other mycotoxins in mothers exposed to water-damaged environments as the data is strong that OTA can be excreted in breast milk and that the consequences of this exposure can be significant especially given the increased susceptibility of the neonate to toxins. For example, rat neonates exposed to OTA in only lactational milk had a 4 to 5 times higher level of OTA compared with those rats exposed only via the placenta [60]. Results of a recent biomonitoring study in Chile confirmed the presence of OTA in breast milk at levels such that the tolerable daily intake could be exceeded [75]. Of concern, in 50 lactating mothers and their infants in Egypt, the presence of OTA was associated with significantly higher levels of urine microglobulin and microalbuminuria in the infants consistent with early renal injury [76]. Moreover, the level of OTA in the infants sera correlated with the degree of microalbuminuria [76], raising great concern about maternal transfer of OTA and injury to the infants.

If elevated levels of urinary OTA are found, there are a number of safe treatments that should be considered to lower the body burden of this mycotoxin. The most important are the avoidance of further exposure and the use of the bile acid resin binding agent, cholestyramine, to decrease enterohepatic recirculation of OTA. Studies have shown that animals fed a diet of OTA plus cholestyramine had a significant shift of OTA from the plasma and urine to the stool, where it is presumably excreted bound to cholestyramine [74]. This will safely reduce the burden on the kidneys as cholestyramine is not absorbed systemically and remains in the gastrointestinal tract. Side effects, which are primarily limited to the gastrointestinal tract, must be considered as well as the timing of cholestyramine away from medications and vitamins, primarily fat soluble vitamins. Many patients tolerate the pure resin better than the commercial prescription prepared with sugar, artificial colors, and a number of additives. As a number of patients with kidney disease also have hyperlipidemia, cholestyramine could potentially beneficial for its lipid lowering effect.

Other potential sequestrant treatments include the use of charcoal which is included in the military textbook recommendations for exposure to trichothecene mycotoxins which has been associated with Yellow Rain exposure [77]. Clay and zeolite have been studied for their efficacy of mycotoxin binding in animals [78, 79] and likely have a use in human illness caused by mycotoxins including OTA.

The use of licorice extract and melatonin was mentioned earlier in this paper and require further study, but may offer a safe option for reducing the toxicity of ochratoxin. There is some evidence that sauna shifts the excretion of ochratoxin to sweat; however, use of sauna needs to be very carefully monitored, especially on initiation. Antioxidants, including glutathione, are also likely to be helpful for their antioxidant and detoxification effects [23]. Vitamins A, C, E and selenium are other potentially beneficial antioxidants that may be protective in their role as superoxide anion scavengers [22, 75].

As we learn more about genomic studies, including the evaluation of cytochrome p450 and glutathione (GSTP and GSTM) pathways, it may be possible to identify those who may be most vulnerable to illness after exposure to ochratoxin and other toxic agents and having this information could be invaluable in directing appropriate therapies and medication use in the future.

 Given the important metabolism of OTA that occurs in the gut, and evidence of increased toxicity when gut flora is disturbed with an antibiotic, it would pay to direct attention toward achieving and maintaining healthy gastrointestinal functioning. In fact, there is evidence that some beneficial gastrointestinal flora can have positive effects on decreasing toxicity from OTA including specific strains of yeast [80]. Interestingly, aspartame has been found to decrease toxicity of OTA through a phenylalanine-mediated mechanism [38]; however, there may be some concerns about other potential toxicities associated with aspartame.

Most importantly, anyone with chronic illness should carefully evaluate their environment and other sources of potential toxic exposures and make every effort to control these exposures. Unfortunately, in the case of exposures to mycotoxins including ochratoxin, it is imperative to address issues of cross contamination of items exposed to water-damaged/mold contaminated environment. Mycotoxins are very difficult to destroy and travel readily on fine, often submicron-sized particles making simple spore testing inadequate for determining the presence of mycotoxins. Thus, a thorough approach is needed to address contamination of items exposed to water-damaged environments to avoid continued exposure to mycotoxins including ochratoxin through these items even if the building is no longer a source of exposure.

## References

[1] Clark HA, Snedeker SM (2006). Ochratoxin A: its cancer risk and potential for exposure. *Journal of Toxicology and Environmental Health B*.

[2] Varga J, Kevel E, Rinyu E, Teren J, Kozakiewicz Z (1996). Ochratoxin production by Aspergillus species. *Applied and Environmental Mircrobiology*.

[3] Larsen TO, Svendsen A, Smedsgaard J (2001). Biochemical characterization of ochratoxin A-producing strains of the genus penicillium. *Applied and Environmental Microbiology*.

[4] Reddy L, Bhoola K (2010). Ochratoxins-food contaminants: impact on human health. *Toxins*.

[5] Kuiper-Goodman T, Scott PM (1989). Risk assessment of the mycotoxin ochratoxin A. *Biomedical and Environmental Sciences*.

[6] Benford D, Boyle C, Dekant W (2001). Ochratoxin A. *JECFA*.

[7] Hagelberg S, Hult K, Fuchs R (1989). Toxicokinetics of ochratoxin A in several species and its plasma-binding properties. *Journal of Applied Toxicology*.

[8] Galtier P (1991). Pharmacokinetics of ochratoxin A in animals. *IARC Scientific Publications*.

[9] Zepnik H, Pahler A, Schauer U, Dekant W (2011). Ochratoxin A induced tumour formation: is there a role of reactive ochratoxin A metabolites?. *Toxicological Sciences*.

[10] Galtier P (1978). Contribution of pharmacokinetic studies to mycotoxicology-Ochratoxin A. *Veterinary Science Communications*.

[11] Madhyastha MS, Marquardt RR, Frohlich AA (1992). Hydrolysis of ochratoxin A by the microbial activity of digesta in the gastrointestinal tract of rats. *Archives of Environmental Contamination and Toxicology*.

[12] Galtier P, Charpenteau JL, Alvinerie M, Labouche C (1979). The pharmacokinetic profile of ochratoxin A in the rat after oral and intravenous administration. *Drug Metabolism and Disposition*.

[13] World Health Organization (1990). *Environmental Health Criterai 105: Selected Mycotoxins: Ochratoxin, Trichothecenes, Ergot*.

[14] Bauer J, Gareis M (1987). Ochratoxin A in the food chain. *Journal of Veterinary Medicine B*.

[15] Gautier JC, Richoz J, Welti DH (2001). Metabolism of ochratoxin A: absence of formation of genotoxic derivatives by human and rat enzymes. *Chemical Research in Toxicology*.

[16] Omar RF, Gelboin HV, Rahimtula AD (1996). Effect of cytochrome P450 induction on the metabolism and toxicity of ochratoxin A. *Biochemical Pharmacology*.

[17] Pfohl-Leszkowicz A, Chakor K, Creppy E, Dirheimer G, Castegnaro M, Plestina R, Dirheimer G, Chernozemsky IN, Bartsch H (1991). DNA adduct formation in mice treated with ochratoxin A. *Mycotoxins, Endemic Nephropathy and Urinary Tract Tumours*.

[18] Pfohl-Leszkowicz A, Grosse Y, Castegnaro M, Castegnaro M, Plestina R, Dirheimer G, Chernozemsky IN, Bartsch H (1993). Ochratoxin A-related DNA adducts in urinary tract tumours of Bulgarian subjects. *Mycotoxins, Endemic Nephropathy and Urinary Tract Tumours*.

[19] Pfohl-Leszkowicz A, Pinelli E, Bartsch H, Mohr U, Castegnaro M (1998). Sex- and strain-specific expression of cytochrome P450s in ochratoxin A- induced genotoxicity and carcinogenicity in rats. *Molecular Carcinogenesis*.

[20] Castegnaro M, Mohr U, Pfuhl-Leszkowicz A (1998). Sex- and strain-specific induction of renal tumors by ochratoxin A in rats correlates with DNA adduction. *International Journal of Cancer*.

[21] Pfohl-Leszkowicz A, Tozlovanu M, Manderville R, Peraica M, Castegnaro M, Stefanovic V (2007). New molecular and field evidences for the implication of mycotoxins but not aristolochic acid in human nephropathy and urinary tract tumor. *Molecular Nutrition and Food Research*.

[22] Grosse Y, Chekir-Ghedira L, Huc A (1997). Retinol, ascorbic acid and *α*-tocopherol prevent DNA adduct formation in mice treated with the mycotoxins ochratoxin A and zearalenone. *Cancer Letters*.

[23] Pfohl-Leszkowicz A, Bartsh H, Azemar B, Mohr U, Esteve J, Castegnaro M (2002). MESNA protects rats against nephrotoxity but not carcinogenicity induced by ochratoxin A implicating two separate pathways. *Medicine and Biology*.

[24] Stemmer K, Ellinger-Ziegelbauer H, Ahr HJ, Dietrich DR (2009). Molecular characterization of preneoplastic lesions provides insight on the development of renal tumors. *American Journal of Pathology*.

[25] Harwig J, Kuiper-Goodman T, Scott PM, Rechcigl M (1983). Microbial food toxicants: ochratoxins. *Handbook of Foodborne Diseases of Biological Origin*.

[26] Mortensen HP, Hald B, Madsen A (1983). Feeding experiments with ochratoxin A contaminated barley for bacon pigs. 5. Ochratoxin A in pig blood. *Acta Agriculturae Scandinavica*.

[27] Madsen A, Mortensen HP, Hald B (1982). Feeding experiments with ochratoxin A contaminated barley for bacon pigs. II. Naturally contaminated barley given for 6 weeks from 20 kg compared with normal barley supplemented with crystalline ochratoxin A and/or citrinin. *Acta Agriculturae Scandinavica A*.

[28] Breitholtz-Emanuelsson A, Fuchs R, Hult K, Appelgren LE (1992). Syntheses of ^14^C-ochratoxin A and ^14^C-ochratoxin B and a comparative study of their distribution in rats using whole body autoradiography. *Pharmacology and Toxicology*.

[29] Micco C, Ambruzzi A, Miraglia M (1991). Contamination of human milk with ochratoxin A. *Mycotoxins Endemic Nephropathy Urinary Tract Tumors*.

[30] Micco C, Miraglia M, Brera C, Corneli S, Ambruzzi A (1995). Evaluation of ochratoxin a level in human milk in Italy. *Food Additives and Contaminants*.

[31] Jonsyn FE, Maxwell SM, Hendrickse RG (1995). Ochratoxin A and aflatoxins in breast milk samples from Sierra Leone. *Mycopathologia*.

[32] Hooper DG Personal communication.

[33] Thrasher J (2012). A water damaged home and health occupants: a case study. *Journal of Environmental and Public Health*.

[34] Moroi K, Suzuki S, Kuga T, Yamazaki M, Kanisawa M (1985). Reduction of ochratoxin A toxicity in mice treated with phenylalanine and phenobarbital. *Toxicology Letters*.

[35] Chakor K, Creppy EE, Dirheimer G (1988). In vivo studies on the relationship between hepatic metabolism and toxicity of ochratoxin A. *Archives of Toxicology*.

[36] Malekinejad H, Farshid A, Mirzakhani N (2011). Liquorice plant extract reduces ochratoxin A-induced nephrotoxicity in rats. *Experimental and Toxicologic Pathology*.

[37] Creppy EE, Schlegel M, Roschenthaler R, Dirheimer G (1980). Phenylalanine prevents acute poisoning by ochratoxin-a in mice. *Toxicology Letters*.

[38] Creppy EE, Baudrimontl AM (1998). How aspartame prevents the toxicity of ochratoxin A. *The Journal of Toxicological Sciences*.

[39] Galtier P, Camguilhem R, Bodin G (1980). Evidence for in vitro and in vivo interaction between ochratoxin A and three acidic drugs. *Food and Cosmetics Toxicology*.

[40] Hooper DG, Bolton VE, Guilford FT, Straus DC (2009). Mycotoxin detection in human samples from patients exposed to environmental molds. *International Journal of Molecular Sciences*.

[41] Wang Y, Chai T, Lu G (2008). Simultaneous detection of airborne Aflatoxin, Ochratoxin and Zearalenone in a poultry house by immunoaffinity clean-up and high-performance liquid chromatography. *Environmental Research*.

[42] Richard JL, Plattner RD, May J, Liska SL (1999). The occurrence of ochratoxin A in dust collected from a problem household. *Mycopathologia*.

[43] Polizzi V, Delmulle B, Adams A (2009). JEM Spotlight: fungi, mycotoxins and microbial volatile organic compounds in mouldy interiors from water-damaged buildings. *Journal of Environmental Monitoring*.

[44] Skaug MA, Eduard W, Størmer FC (2001). Ochratoxin A in airborne dust and fungal conidia. *Mycopathologia*.

[45] Breitholtz-Emanuelsson A, Fuchs R, Hult K (1995). Toxicokinetics of ochratoxin A in rat following intratracheal administration. *Natural Toxins*.

[46] Dipaolo N, Guarnieri A, Garosi G, Sacchi G, Mangiarotti AM (1994). Inhaled mycotoxins lead to acute renal failure. *Nephrology Dialysis Transplantation*.

[47] Genuis Personal Communication.

[48] Pfohl-Leszkowicz A, Manderville RA (2007). Ochratoxin A: an overview on toxicity and carcinogenicity in animals and humans. *Molecular Nutrition and Food Research*.

[49] Bendele AM, Carlton WW, Krogh P, Lillehoj EB (1985). Ochratoxin A carcinogenesis in the (C57BL/6J x C3H)F1 mouse. *Journal of the National Cancer Institute*.

[50] Huff JE, Castegnaro M, Plestina R, Dirheimer G, Chemozemsky IN, Bartsch H (1991). Carcinogenicity of ochratoxin A in experimental animals. *Mycotoxins, Endemic Nephropathy and Urinary Tract Tumors*.

[51] IARC (1993). Ochratoxin A. *IARC Working Group on the Evaluation of Carcinogenic Risks to Humans; Some Naturally Occurring Substances. Food Items and Constituents, Heterocyclic Aromatic Amines and Mycotoxins*.

[52] National Toxicology Program (1989). *Toxicology and Carcinogenesis Studies of Ochratoxin A (CAS no. 303-47-9) ub F344/N rats (Gavage Studies)*.

[53] Gekle M, Silbernagl S (1994). The role of the proximal tubule in ochratoxin A nephrotoxicity in vivo: toxodynamic and toxokinetic aspects. *Renal Physiology and Biochemistry*.

[54] Gekle M, Oberleithner H, Silbernagl S (1993). Ochratoxin A impairs ’postproximal’ nephron function in vivo and blocks plasma membrane anion conductance in Madin-Darby canine kidney cells in vitro. *Pflugers Archiv European Journal of Physiology*.

[55] Desalegn B, Nanayakkara S, Harada KH (2011). Mycotoxin detection in urine samples from patients with chronic kidney disease of uncertain etiology in Sri Lanka. *Bulletin of Environmental Contamination and Toxicology*.

[56] Schwartz GG (2002). Hypothesis: does ochratoxin A cause testicular cancer?. *Cancer Causes and Control*.

[57] Zaied C, Bouaziz C, Azizi I (2011). Presence of ochratoxin A in Tunisian blood nephropathy patients. Exposure level to OTA. *Experimental and Toxicologic Pathology*.

[58] Maaroufi K, Achour A, Hammami M (1995). Ochratoxin A in human blood in relation to nephropathy in Tunisia. *Human and Experimental Toxicology*.

[59] Appelgren LE, Arora RG (1983). Distribution of ^14^C-labelled ochratoxin A in pregnant mice. *Food and Chemical Toxicology*.

[60] Hallen IP, Breitholtz-Emanuelsson A, Hult K, Olsen M, Oskarsson A (1998). Placental and lactational transfer of ochratoxin A in rats. *Natural Toxins*.

[61] Ferrufino-Guardia EV, Tangni EK, Larondelle Y, Ponchaut S (2000). Transfer of ochratoxin A during lactation: exposure of suckling via the milli of rabbit does fed a naturally-contaminated feed. *Food Additives and Contaminants*.

[62] Skaug MA, Helland I, Solvoll K, Saugstad OD (2001). Presence of ochratoxin A in human milk in relation to dietary intake. *Food Additives and Contaminants*.

[63] Gürbay A, Girgin G, Sabuncuoğlu SA (2010). Ochratoxin A: is it present in breast milk samples obtained from mothers from Ankara, Turkey?. *Journal of Applied Toxicology*.

[64] Zimmerli B, Dick R (1995). Determination of ochratoxin A at the ppt level in human blood, serum, milk and some foodstuffs by high-performance liquid chromatography with enhanced fluorescence detection and immunoaffinity column cleanup: methodology and Swiss data. *Journal of Chromatography B*.

[65] Fenske M, Fink-Gremmels J (1990). Effects of fungal metabolites on testosterone secretion in vitro. *Archives of Toxicology*.

[66] Zhang X, Boesch-Saadatmandi C, Lou Y, Wolffram S, Huebbe P, Rimbach G (2009). Ochratoxin A induces apoptosis in neuronal cells. *Genes and Nutrition*.

[67] Belmadani A, Steyn PS, Tramu G, Betbeder AM, Baudrimont I, Creppy EE (1999). Selective toxicity of ochratoxin a in primary cultures from different brain regions. *Archives of Toxicology*.

[68] Sava V, Velasquez A, Song S, Sanchez-Ramos J (2007). Adult hippocampal neural stem/progenitor cells in vitro are vulnerable to the mycotoxin ochratoxin-A. *Toxicological Sciences*.

[69] Sava V, Reunova O, Velasquez A, Harbison R, Sánchez-Ramos J (2006). Acute neurotoxic effects of the fungal metabolite ochratoxin-A. *NeuroToxicology*.

[70] Al-Anati L, Petzinger E (2006). Immunotoxic activity of ochratoxin A. *Journal of Veterinary Pharmacology and Therapeutics*.

[71] Montagnoli C, Fallarino F, Gaziano R (2006). Immunity and tolerance to Aspergillus involve functionally distinct regulatory T cells and tryptophan catabolism. *Journal of Immunology*.

[72] Rao TKS, Soman A, Nzerue CM Focal segmental glomeruloscerosis. http://emedicine.medscape.com/article/245915-overview.

[73] Canaud G, Dion D, Zuber J (2010). Recurrence of nephritic syndrome after transplantation in a mixed population of children and adults: course of glomerular lesions and value of the Columbia classification of histological variants of focal ad segmental glomerulosclerosis (FSGS). *Nephrology Dialysis Transplantation*.

[74] Kerkadi A, Barriault C, Tuchweber B (1998). Dietary cholestyramine reduces ochratoxin A-induced nephrotoxicity in the rat by decreasing plasma levels and enhancing fecal excretion of the toxin. *Journal of Toxicology and Environmental Health A*.

[75] Muñoz K, Campos V, Blaszkewicz M (2010). Exposure of neonates to ochratoxin A: first biomonitoring results in human milk (colostrum) from Chile. *Mycotoxin Research*.

[76] Hassan AM, Sheashaa HA, Fattah MFA, Ibrahim AZ, Gaber OA, Sobh MA (2006). Study of ochratoxin A as an environmental risk that causes renal injury in breast-fed Egyptian infants. *Pediatric Nephrology*.

[77] Wannemacher R, Weiner S (1998). Chapter 34- trichothecene mycotoxins in the textbook. *Medical Aspects of Chemical and Biological Warfare*.

[78] Piva A, Galvano F Nutritional approaches to reduce the impact of mycotoxins. http://www.engormix.com/.

[79] Whitlow L Evaluation of mycotoxin binders.

[80] Schatzmayr G, Zehner F, Täubel M (2006). Microbiologicals for deactivating mycotoxins. *Molecular Nutrition and Food Research*.

